# CT-Based Radiomics Signature With Machine Learning Predicts MYCN Amplification in Pediatric Abdominal Neuroblastoma

**DOI:** 10.3389/fonc.2021.687884

**Published:** 2021-05-24

**Authors:** Xin Chen, Haoru Wang, Kaiping Huang, Huan Liu, Hao Ding, Li Zhang, Ting Zhang, Wenqing Yu, Ling He

**Affiliations:** ^1^ Department of Radiology, Children’s Hospital of Chongqing Medical University, National Clinical Research Center for Child Health and Disorders, Ministry of Education Key Laboratory of Child Development and Disorders, Chongqing Key Laboratory of Pediatrics, Chongqing, China; ^2^ GE Healthcare, Shanghai, China

**Keywords:** children, abdomen, neuroblastoma, MYCN, radiomics, prediction

## Abstract

**Purpose:**

MYCN amplification plays a critical role in defining high-risk subgroup of patients with neuroblastoma. We aimed to develop and validate the CT-based machine learning models for predicting MYCN amplification in pediatric abdominal neuroblastoma.

**Methods:**

A total of 172 patients with MYCN amplified (n = 47) and non-amplified (n = 125) were enrolled. The cohort was randomly stratified sampling into training and testing groups. Clinicopathological parameters and radiographic features were selected to construct the clinical predictive model. The regions of interest (ROIs) were segmented on three-phrase CT images to extract first-, second- and higher-order radiomics features. The ICCs, mRMR and LASSO methods were used for dimensionality reduction. The selected features from the training group were used to establish radiomics models using Logistic regression, Support Vector Machine (SVM), Bayes and Random Forest methods. The performance of four different radiomics models was evaluated according to the area under the receiver operator characteristic (ROC) curve (AUC), and then compared by Delong test. The nomogram incorporated of clinicopathological parameters, radiographic features and radiomics signature was developed through multivariate logistic regression. Finally, the predictive performance of the clinical model, radiomics models, and nomogram was evaluated in both training and testing groups.

**Results:**

In total, 1,218 radiomics features were extracted from the ROIs on three-phrase CT images, and then 14 optimal features, including one original first-order feature and eight wavelet-transformed features and five LoG-transformed features, were identified and selected to construct the radiomics models. In the training group, the AUC of the Logistic, SVM, Bayes and Random Forest model was 0.940, 0.940, 0.780 and 0.927, respectively, and the corresponding AUC in the testing group was 0.909, 0.909, 0.729, 0.851, respectively. There was no significant difference among the Logistic, SVM and Random Forest model, but all better than the Bayes model (p <0.005). The predictive performance of the Logistic radiomics model based on three-phrase is similar to nomogram, but both better than the clinical model and radiomics model based on single venous phase.

**Conclusion:**

The CT-based radiomics signature is able to predict MYCN amplification of pediatric abdominal NB with high accuracy based on SVM, Logistic and Random Forest classifiers, while Bayes classifier yields lower predictive performance. When combined with clinical and radiographic qualitative features, the clinics-radiomics nomogram can improve the performance of predicting MYCN amplification.

## Introduction

Neuroblastoma (NB) is one of the most common solid malignancy in children originating from neural crest tissues along the sympathetic chains (1). NB can arise from various anatomical compartments (i.e., neck, chest, abdomen or pelvis), but most frequently arise from the abdomen (adrenal gland or extra-adrenal retroperitoneum), accounting for 73% of all systems (2). As a kind of heterogeneous tumor, the clinical outcome of abdominal NB varies from spontaneous regression to extensive systemic metastasis (3). For the pediatric patients with abdominal NB in advanced stage, the long-term survival rate is less than 50% regardless of the intensive treatment (4). Therefore, risk stratification is vital enough to choose the optimal therapy for individuals in the era of precision medicine (5). Among the various attempts from different international groups aimed to identify factors that can be used to risk stratification and to define an sub-population with poor clinical outcome (6), all groups highlight the significance of MYCN amplification status for defining high-risk group and consider that all patients with MYCN amplified are prone to relapse (2). Clinically, the amplification of MYCN oncogene is significantly correlated to an aggressive phenotype (7). Therefore, the detection of MYCN amplification status is critical to risk-stratify patients. However, as an invasive method, traditional biopsy may cause various complications (8). Meanwhile, the availability of detection of MYCN has been hindered by the limited access to genetic testing methods in many institutions (9), therefore, an alternative non-invasive method is needed to characterize the MYCN amplification status availably.

In recent years, the increasing application of radiomics in solid tumors has resulted in the emergence of radiogenomics. The heart of radiogenomics is to identify and predict the expression of clinically significant molecular biomarkers of tumors by analyzing high-dimensional quantitative signatures extracted from tumor regions of interest (ROIs) in radiographic images (9, 10). Compared with histopathology and genetic testing methods, radiogenomics not only can overcome sampling bias and the possible complications caused by biopsy, but also is expected to provide more comprehensive and accurate information in predicting the biomarkers (9). To date, the application of radiogenomics in pediatric tumors is mainly focused on MRI-based signatures of medulloblastoma, and the CT-based radiogenomics is rarely used (11, 12). Although a recent study has shown the potential of CT-based signature in the prediction of MYCN amplification of NB and ganglioneuroblastoma (GNB) (8), there were some problems with the patients’ selection, in which nonabdominal NB and GNB were also enrolled, because previous literatures have demonstrated that MYCN amplification rarely occurs in nonabdominal NB and GNB (13, 14). Meanwhile, due to the heterogeneity of NB, the ROIs selectively delineated on several largest levels of the tumor cannot reflect the biological characteristics of the tumor comprehensively (15). Instead, the whole-tumor ROIs delineated on all slices in other radiomics studies have contributed to reduce sampling bias and improve intra- and inter-observer consistency (16, 17).

In the present study, we developed and validated the CT-based radiomics features combined with various machine learning methods for predicting MYCN amplification of abdominal NB in the cohort of pediatric patients. Besides, we constructed a clinical model based on clinicopathological parameters and radiographic features, and then added the radiomics signature to develop radiomics-clinics model. The predictive performance of clinical model, radiomics model and radiomics-clinics model was finally evaluated and compared according to the AUC and Delong test.

## Materials and Methods

### Patients and MYCN Amplification Characterization

**Figure 1:**
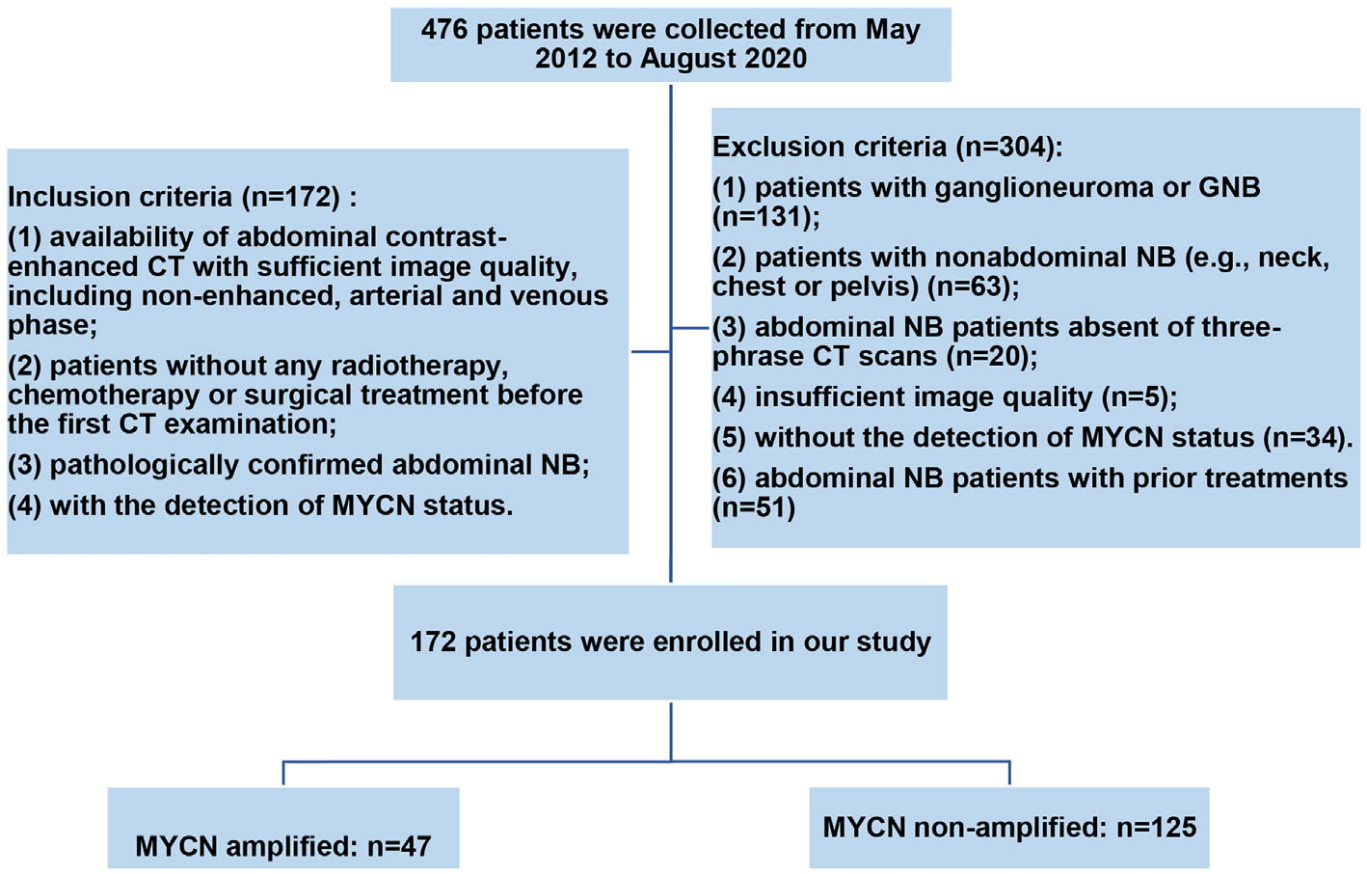
The workflow of patients’ selection in our study.

The Ethics Committee of our hospital approved this single-center retrospective study and waived the requirement for informed patient consent. We identified 172 abdominal NB patients with MYCN amplified (n = 47) and non-amplified (n = 125) by searching the medical record management system and radiology picture archiving and communication system (PACS) of our department from May 2012 to August 2020 consecutively according to the inclusion and exclusion criteria. Inclusion criteria were: (1) availability of abdominal contrast-enhanced CT with sufficient image quality, including non-enhanced, arterial and venous phase; (2) patients without any radiotherapy, chemotherapy or surgical treatment before the first CT examination; (3) pathologically confirmed abdominal NB; (4) with the detection of MYCN status. Exclusion criteria were: (1) patients with ganglioneuroma or GNB; (2) patients with nonabdominal NB (e.g., neck, chest or pelvis); (3) abdominal NB patients absent of three-phrase CT scans; (4) insufficient image quality; (5) without the detection of MYCN status; (6) abdominal NB patients with prior treatments. The detailed workflow of patients’ selection is shown in **Figure 1**.

The study cohort was randomly stratified sampling into training group and testing group in a proportion of 7:3. Clinicopathological parameters, including gender, age (month), histopathology, INSS stage, Shimada classification and urinary vanillylmandelic acid (VMA) were collected from medical records. According to the differentiation degree, the histopathological results were categorized into two groups: undifferentiated or poorly differentiated, and differentiated NB (8). The prognostic Shimada classification of patients was defined as favorable histology (FH) and unfavorable histology (UFH) on the basis of age, degree of differentiation and mitotic karyorrhectic index (MKI) of NB (18). The MYCN gene copy number was detected by fluorescence *in situ* hybridization (FISH) method in all specimens using a MYC-N/LAF double probe, and cases with the number of signals exceeding 10-fold MYCN copies were considered to be MYCN amplified (18). The intervals between the MYCN status detection and contrast-enhanced CT scans of the same patient in the present study were less than a month.

### CT Scanning

All CT scans were acquired during a single breath-hold in cooperative children or during quiet respiration in children unable to suspend respiration, and those who could not cooperate were sedate by oral administration of 10% chloral hydrate (0.5 ml/kg, body weight) before examination. All abdominal three-phase CT scans, including non-enhanced phase (NP), arterial phase (AP), and venous phase (VP), were performed on Lightspeed VCT 64-slice spiral CT (GE Healthcare, USA) scanner or Brilliance ICT 256-slice spiral CT (Philips, Netherlands) scanner. The CT scanning parameters were (1) tube voltage: 120 kV; (2) tube current: 200 mAs; (3) pitch: 0.984:1; (4) slice thickness: 5.0 mm; (5) slice interval: 5.0 mm; (6) reconstructed slice thickness: 1.25 mm. Nonionic iodinated contrast material (Omnipaque 300 mg I/mL or Visipaque 320 mg I/ml, GE Healthcare) was used. Contrast material (2 ml/kg, body weight) was injected into peripheral vein of the forearm with a power injector at a rate of 1–3 ml/s. AP and VP of post-contrast scanning were performed at 20–35 and 60–70 s respectively after contrast material administration.

### Imaging Analysis

All CT examinations were transmitted to the workstation for review and analysis. All images were initially analyzed independently by two experienced pediatric radiologists without knowledge of the MYCN status. The tumor features, including calcification (present or not), infiltrating across midline (exceeding the contralateral edge of the spine, present or not) and necrosis (present or not), were recorded[8]. Disagreements were resolved by negotiation.

### Clinical Model Building

Clinicopathological parameters of MYCN-amplified and non-amplified groups included gender, age, histopathology, INSS stage, Shimada classification and urinary vanillylmandelic acid (VMA) and radiographic features. Influence characteristics that were statistically significant with p<0.05 in the univariate logistic analyses were included in the multivariate analysis following the stepwise selection method. The Akaike information criterion (AIC) and Log-Likelihood were used as the stopping rules to select the most predictive clinical features.

### Image Preprocessing and Tumor Segmentation

**Figure 2:**
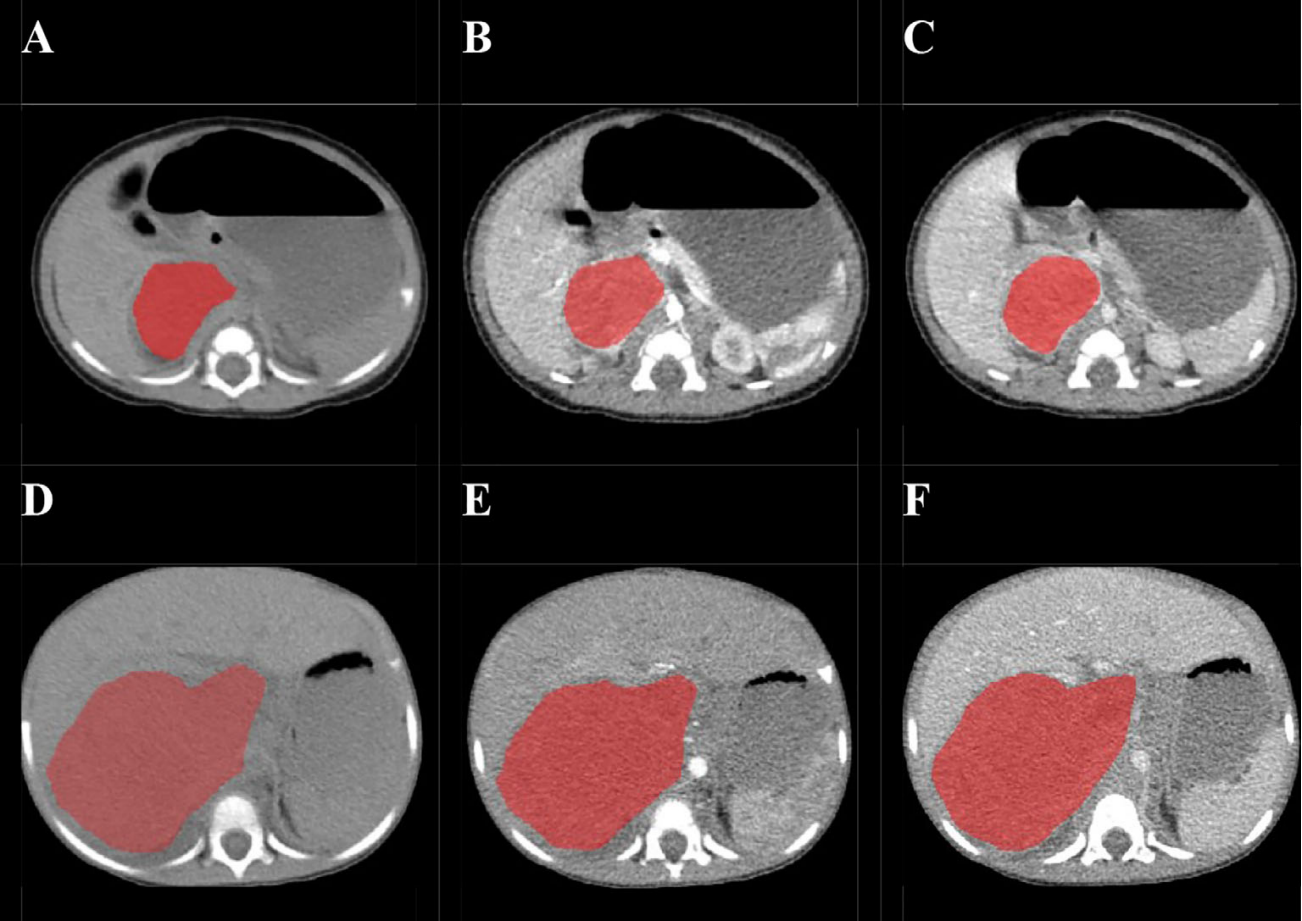
Examples of manual delineated regions of interests (ROIs) of amplified and non-amplified NB. Delineation of the ROI on one slice of a non-amplified NB (Female, 2 months, asymptomatic) on non-enhanced phase (NP) **(A)**, arterial phase (AP) **(B)**, and venous phase (VP) **(C)**; Delineation of the ROI on one slice of an amplified NB (female, 2 years, presented with abdominal palpable mass) on NP **(D)**, AP **(E)**, and VP **(F)**.

Before the tumor segmentation, isotropic voxel resampling into 1 mm × 1 mm × 1 mm with linear interpolation was used to image preprocessing for purpose of normalizing the geometry of CT images. The ROIs of whole-tumor were manually 3D-delineated on three phrases respectively using a free open-source software package (ITK-SNAP, ver.3.4.0) by a pediatric radiologist with 2 years of experience, and the ROIs were then reviewed and confirmed by the other pediatric radiologist with 10 years of experience (**Figure 2**). The ROIs included the calcification and necrosis area of the lesion. The ROIs segmentation of each tumor was performed twice by reader 1 (time-interval of 2 weeks) and once by reader 2. The intra-observer class correlation coefficients (ICCs) were calculated based on the features extracted from the ROIs delineated by reader 1 at different time points.

### Radiomics Features Extraction and Selection

The images and corresponding ROIs were imported into the in-house software (Artificial Intelligence Kit, AK, Version V3.2.2.R, GE Healthcare) together, and then features extraction was performed with AK software. The radiomics features were classified into seven groups including: first order, shape, gray-level co-occurrence matrix (GLCM), gray-level size-zone matrix (GLSZM), gray-level run-length matrix (GLRLM), neighborhood gray-tone difference matrix (NGTDM) and neighboring gray-level dependence matrix (GLDM). To enhance intricate patterns in the data invisible to the human eye, advanced filters, including Laplacian of Gaussian (LoG; sigma, 2.0 and 3.0 mm), and wavelet decompositions with all possible combinations of high (H) or low (L) pass filter in each of the three dimensions (HHH, HHL, HLH, LHH, LLL, LLH, LHL, HLL), were applied. A total of 3654 radiomic features of each patient (1218 features in each phase) were finally extracted from the ROIs based on NP, AP and VP.

Because many of the extracted high-dimensional features are often redundant and meaningless, a variety of methods were used for dimensionality reduction. To begin with, intra-observer analysis was used to assess the reliability and reproducibility of the features in order to find out the robust features. Features with ICCs higher than 0.80 were considered reliable and selected. Then, two feature selection methods, the maximum relevance minimum redundancy (mRMR) and the least absolute shrinkage and selection operator (LASSO) regression, were applied to eliminate the redundant and irrelevant features and choose the optimized subset of features to construct the radiomics models. Due to the CT scans in our study were performed on two scanners from different manufacturers (Lightspeed VCT and Brilliance ICT), the performance of radiomics features derived from two scanners was evaluated by ROC analysis and Delong test. Rad-score was calculated by summing the selected features weighted by their coefficients.

### Machine Learning

The selected features from the training group were used to establish radiomics models based on three-phrase using Logistic regression, Support Vector Machine (SVM), Bayes and Random Forest. The performance of the developed radiomics models were then validated in both training and testing groups according to the area under the receiver operator characteristic (ROC) curve (AUC). The Delong test was used to compare the performance of four different machine learning models.

### Nomogram Building and Evaluating

Finally, the radiomics signature was added to build the radiomics-clinics nomogram incorporated of statistically significant clinicopathological parameters and radiographic features on the basis of the results of multivariate logistic regression analysis in the training group. The predictive performance of the clinical model, radiomics models, and nomogram was evaluated according to the area under the receiver operator characteristic (ROC) curve (AUC) in both training and testing groups, and the Delong test was applied to compare the performance of different models.

### Statistical Analysis

IPM statistics (IPMs, version 2.4.0, GE healthcare) and R programming language (ver. 3.4.2, http://www.r-project.org) were used to carry out statistical analysis. A chi square test or Fisher’s exact test was used for the nominal variables, and a Mann–Whitney test was used for the continuous variables with abnormal distribution between the two cohorts. A two-tailed p <0.05 indicated statistical significance. “mRMRe” and “glmnet” packages were used to carry out the mRMR and LASSO respectively. The “pROC” package was used to perform Delong test and plot the ROC curves of each model. The “rms” package was used to carry out machine learning and build clinical-radiomics nomogram.

## Results

### Patient Characteristics and Clinical Model Building

According to the inclusion and exclusion criteria, 172 patients were identified in the present study (47 patients with MYCN amplified and 125 patients with MYCN non-amplified). The patients were divided into training group (n = 121) and testing group (n = 51) randomly in a proportion of 7:3, and the characteristics of patients are detailed in **Table 1**. The meaningful characteristics, including the INSS stage, Shimada classification, infiltrating across midline, calcification, necrosis and VMA, were identified as significant with p <0.05 by univariate analyzing. Among them, four characteristics, including Shimada classification (odds ratio (OR) = −2.22, p <0.001), infiltrating across midline (OR = 0.89, p = 0.352), calcification (OR = −1.363, p = 0.0017) and VMA (OR = −0.019, p = 0.0127) were selected using the stepwise selection by multivariate logistic regression analysis (**Table 2**). The AIC criterion was in the multivariate analysis following the stepwise selection method, and the model with smallest AIC value would be chosen. The AIC value of the selected model was 171.4549.

**Table 1:** Clinicopathologic and radiographic features in training and testing groups.

Features	Training group (n = 121)			Testing group (n = 51)		
	Non-amplified	Amplified	p-value	Non-amplified	Amplified	p-value
Gender (%)			0.736			0.543
Male	51 (57.95)	18 (54.55)		22 (59.46)	7 (50.00)	
Female	37 (42.05)	15 (45.45)		15 (40.54)	7 (50.00)	
Histopathological types (%)			0.629			0.772
Differentiated	28 (31.82)	9 (27.27)		14 (37.84)	4 (28.57)	
Undifferentiated or poorly differentiated	60 (68.18)	24 (72.73)		23 (62.16)	10 (71.43)	
INSS ^1^ (%)			0.023*			0.43
1	12 (13.64)	0 (0.00)		6 (16.22)	0 (0.00)	
2	7 (7.95)	1 (3.03)		1 (2.70)	0 (0.00)	
3	18 (20.45)	4 (12.12)		8 (21.62)	3 (21.43)	
4	48 (54.55)	28 (84.85)		20 (54.05)	11 (78.57)	
4S	3 (3.41)	0 (0.00)		2 (5.41)	0 (0.00)	
Shimada classification (%)			0.006*			0.071
UFH	58 (65.91)	30 (90.91)		23 (62.16)	13 (92.86)	
FH	30 (34.09)	3 (9.09)		14 (37.84)	1 (7.14)	
Infiltrating across midline (%)			0.17			0.201
Yes	41 (46.59)	20 (60.61)		23 (62.16)	12 (85.71)	
No	47 (53.41)	13 (39.39)		14 (37.84)	2 (14.29)	
Calcification (%)			0.016*			0.454
Yes	61 (69.32)	15 (45.45)		27 (72.97)	8 (57.14)	
No	27 (30.68)	18 (54.55)		10 (27.03)	6 (42.86)	
Necrosis (%)			0.034*			0.099
Yes	51 (57.95)	26 (78.79)		24 (64.86)	13 (92.86)	
No	37 (42.05)	7 (21.21)		13 (35.14)	1 (7.14%)	
Age (P_25_, P_75_)	24.0 (8.45, 48.0)	25.0 (12.0, 40.3)	0.518	15.00 (7.70, 48.00)	22.0 (13.9, 30.3)	0.598
VMA (P_25_, P_75_)	21.7 (5.97, 43.36)	5.14 (2.96, 21.76)	0.005*	21.19 (6.39, 41.27)	4.67 (2.36, 11.6)	0.002*

*p <0.05. A chi square test or Fisher’s exact test was used for the nominal variable. A Mann–Whitney test was used for the continuous variable with abnormal distribution. ^1^ INSS, International Neuroblastoma Stage System. Since the morphological characteristics of lesions were firstly interpreted by two radiologists independently and then the difference was resolved by negotiation, the reader agreements were evaluated [Infiltrating across midline: 0.909 (0.878–0.933), Calcification: 0.871 (0.809–0.909), Necrosis: 0.920 (0.890–0.942)].

**Table 2:** Univariate and Multivariate logistic analysis in the cohort.

Variable	Univariate analysis		Multivariate analysis	
	OR (95%CI)	P-value	OR (95%CI)	P-value
Gender	0.809 (0.412, 1.589)	0.539		
Histopathological type	1.323 (0.632, 2.771)	0.457		
INSS stage	2.040 (1.255-3.316)	0.004*		
Shimada classification	0.171 (0.058, 0.508)	0.001*	−2.22 (−3.396 to −1.045)	<0.001*
Infiltrating across midline	2.033 (1.003, 4.121)	0.049*	0.890 (0.062–1.718)	0.0352*
Calcification	0.403 (0.202, 0.802)	0.010*	−1.363 (−2.212 to −0.513)	0.0017*
Necrosis	3.250 (1.402, 7.533)	0.006*		
Age	0.996 (0.984, 1.008)	0.467		
VMA	0.981 (0.967, 0.996)	0.013*	−0.019 (−0.035 to −0.004)	0.0127*

Intercept = 0.24026. *reflected the significant difference with the P value <0.05.

### Feature Selection and Machine Learning

**Figure 3:**
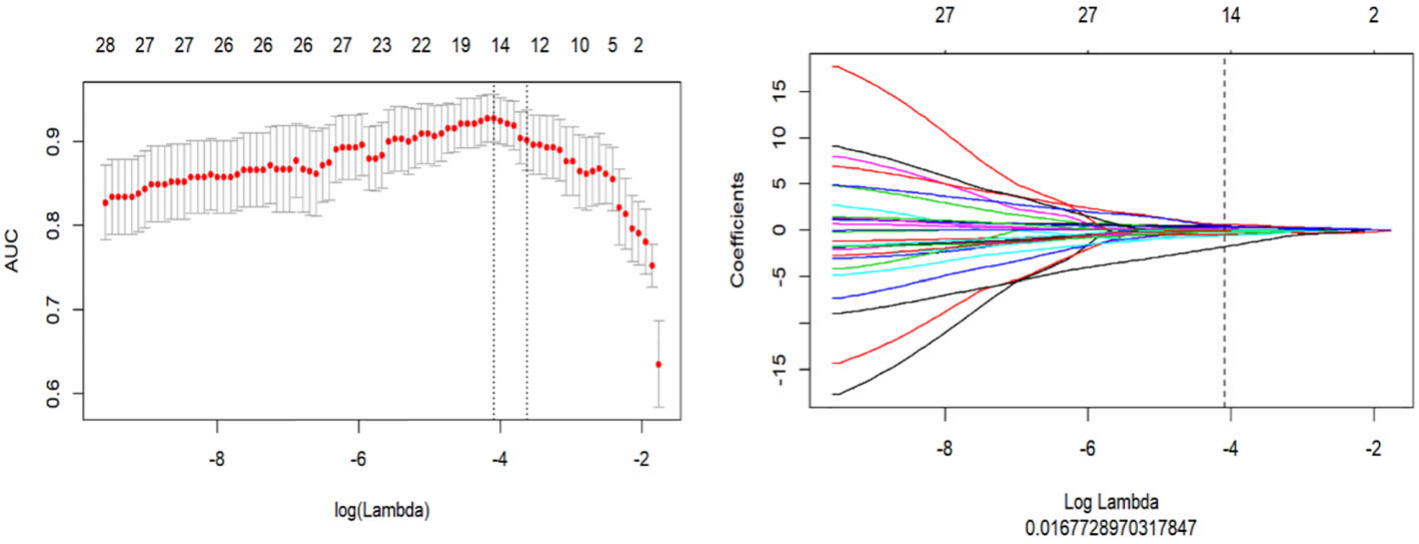
The LASSO includes choosing the regular parameter λ, determining the number of the feature.

**Figure 4:**
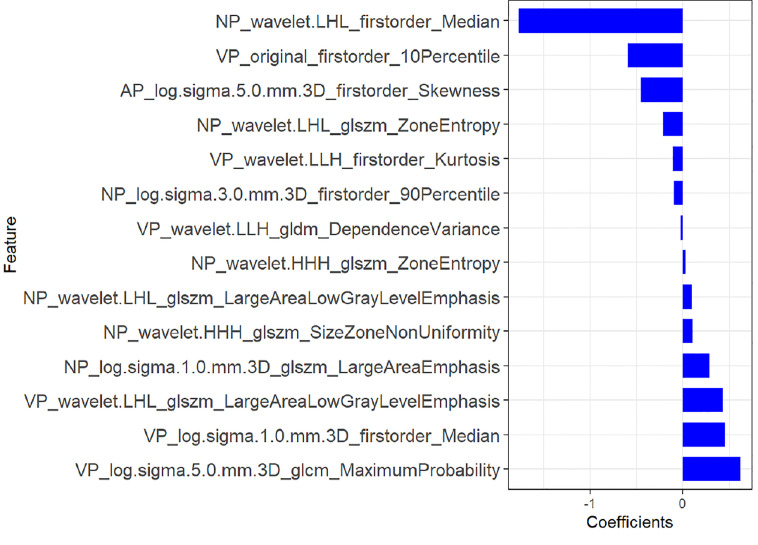
The most predictive subset of feature was chosen and the corresponding coefficients were evaluated in the training group.

A total of 1,218 radiomics features were automatically extracted for each segmented ROI (NP, AP and VP). 734 features were firstly selected with ICCs higher than 0.80 by intra-observer analysis. Before selection of the 734 features, the abnormal or missing values were replaced by the median, and features standardization was applied. And then, mRMR and LASSO were used to select the most optimal features. After the redundant and irrelevant features were removed by mRMR, 30 features from AP, NP and VP were retained. Then LASSO was conducted to identify the final 14 optimal features, including one first-order feature and eight wavelet-transformed features and five LoG-transformed features, to construct the radiomics models. The LASSO includes choosing the regular parameter λ and determining the number of the feature (**Figure 3**). After the number of features determined, the most predictive subset of features was chosen and the corresponding coefficients were calculated (**Figure 4**). The comparison of radiomics signatures derived from two scanners is shown in **Supplementary Figure 1** and **Table 1**, and the performance of the signatures from two scanners was different. Rad-score was calculated by summing the selected features weighted by their coefficients. The final formula of rad-score is showed in **Supplementary Figure 2**.

**Figure 5:**
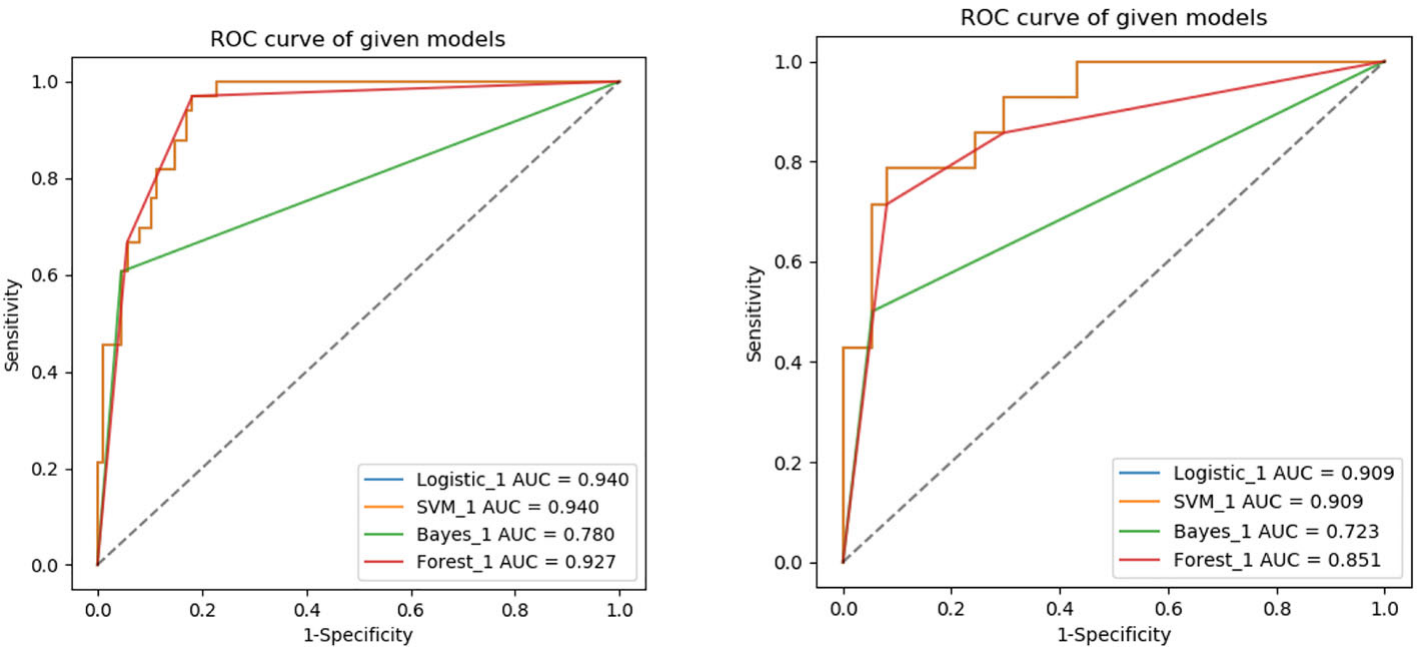
ROC analysis used to evaluate the predictive performance of different radiomics models in the training and testing groups.

The ROC curves of the four machine learning models in the training and testing groups are shown in **Figure 5**. In the training group, the AUC among the Logistic, SVM, Bayes and Random Forest was 0.940, 0.940, 0.780 and 0.927, respectively, and the corresponding AUC in the testing group was 0.909, 0.909, 0.729, 0.851, respectively. The Delong test was applied to compare the performance of the four models. There was no significant difference among the Logistic, SVM and Random Forest model (Logistics vs SVM: p = 0.99, Logistic vs Random Forest: p = 0.33, SVM vs Random Forest: p = 0.33), but all better than the Bayes model (p <0.005) (**Table 3**).

**Table 3:** Comparison of four different machine learning model based on three-phrase in the training and testing groups.

Models	Training group (n = 121)		Validation group (n = 51)	
	AUC (95%CI)	Delong test	AUC (95%CI)	Delong test
Logistic	0.940 (0.901–0.978)	0.99^#^	0.909 (0.824–0.994)	0.99^#^
SVM	0.940 (0.901–0.978)	<0.005^##^	0.909 (0.824–0.994)	<0.005^##^
Bayes	0.780 (0.692–0.867)	<0.005^###^	0.729 (0.581–0.863)	0.051^###^
Forest	0.927 (0.879–0.974)	0.33^####^	0.851 (0.725–0.976)	0.055^####^

^#^indicated the Delong test between the logistic and SVM model; ^##^indicated the DeLong test between the SVM and Bayes model; ^###^indicated the Delong test between the Bayes and Forest model; ^####^indicated the DeLong test between the Logistic and Forest model.

### Nomogram Building and Evaluating

**Figure 6:**
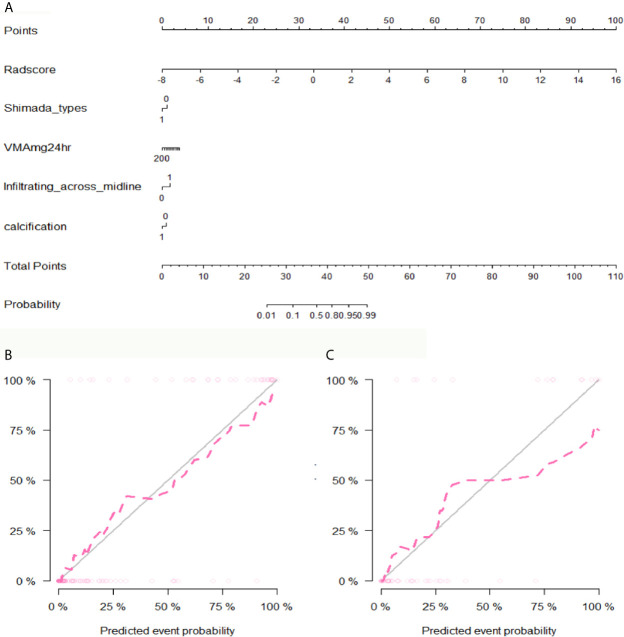
The developed nomogram and calibration curve. **(A)** The nomogram was developed in the training group and incorporated the radiomics signature, Shimada types, VMA, Infiltrating across midline and calcification. **(B)** Calibration plots of the nomogram in the training group. **(C)** Calibration plots of the nomogram in the testing group.

**Figure 7:**
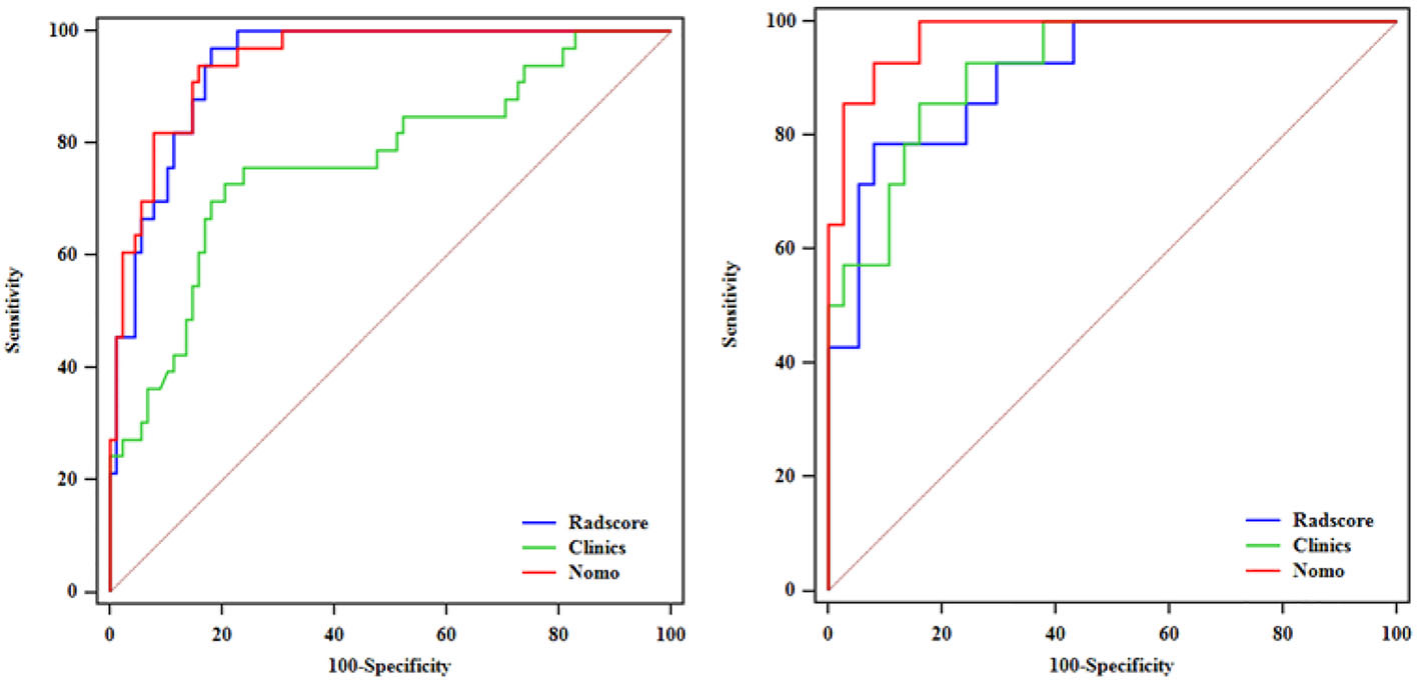
ROC analysis among the clinical model, radiomics model and nomogram in the training and testing groups.

After performing multivariate logistic regression analysis, the radiomics-clinics nomogram was built by incorporating of clinical-radiological predictors (Shimada classification (odds ratio (OR) = −2.22, p <0.001), infiltrating across midline (OR = 0.89, p = 0.352), calcification (odds ratio (OR) = −1.363, p = 0.0017), VMA (odds ratio (OR) = −0.019, p = 0.0127)) (detailed in **Figure 6**) and the calculated radscore. The ROC analysis of the clinical model, radiomics model, and nomogram is illustrated in **Figure 7** and the comparison of different models is shown in **Table 4**. The nomogram had a superior predictive performance than using the clinical model alone, accompanied with an improved AUC value from 0.770 to 0.946 in the training group and 0.917 to 0.977 in the testing group. The performance of the Logistic radiomics model based on three-phrase is similar to nomogram, but both better than clinical model and radiomics model based on single venous phase (**Table 4**).

**Table 4:** Comparison of different models in the training and testing groups.

Variable	Training group				Testing group			
	AUC (95%CI)	Sensitivity	Specificity	Delong	AUC (95%CI)	Sensitivity	Specificity	Delong
VP	0.89 (0.83–0.95)	0.569	0.942		0.88 (0.79–0.97)	0.416	0.852	
Three-phase	0.940 (0.901–0.978)	0.970	0.818	0.0008^#^	0.909 (0.824–0.994)	0.786	0.919	0.884^#^
Clinics	0.770 (0.685–0.841)	0.727	0.795	0.0003^##^	0.917 (0.805–0.976)	0.857	0.834	0.0784^##^
Nomo	0.946 (0.889–0.979)	0.939	0.841	0.343^###^	0.977 (0.89–0.999)	0.928	0.918	0.05^###^

^#^indicated the Delong test between the venous and three-phrase radiomics model; ^##^indicated the Delong test between the three-phrase radiomics and Clinics model; ^###^indicated the Delong test between the three-phrase radiomics and Nomo combined model.

## Discussion

MYCN amplification status plays a significant role in risk classification of NBs, and NBs with MYCN amplified are usually classified into the high-risk group, where the patients need intensive treatment of operation, radiotherapy and chemotherapy (19). In addition to genetic testing method, radiogenomics, which focusing on establishing the correlation between imaging features and molecular biomarkers, is expected to provide an alternative method to characterize and predict the MYCN amplification status of neuroblastoma noninvasively and inexpensively (8, 20). Previous studies on radiogenomics have demonstrated its potential to predict mutated genes in the solid tumors (11, 12, 20, 21). Among adult tumors, CT-based radiogenomics has been widely studied in lung, kidney and liver neoplasms (22–24). However, there have been a few reports on CT radiogenomics in pediatric tumors (8). In this study, data from clinicopathologic parameters (Shimada classification, VMA) and radiographic features (infiltrating across midline, calcification and radiomics features) were selected to develop predictive models for the MYCN amplification of pediatric abdominal NB. Compared to the other study of CT radiogenomics in pediatric NB and GNB (8), we only enrolled the pediatric patients with abdominal NB, because MYCN amplification mostly occurs in abdominal NB (13, 14). Meanwhile, we delineated the whole-tumor ROIs on all slices for the purpose of improving intra- and inter-observer consistency. In addition to first-order and textural features, higher-order features transformed by wavelet and LoG were also extracted to further evaluate the optimal radiomics features correlating with MYCN amplification. Moreover, we also compared the performance of radiomics models developed by four common machine learning methods.

In the present study, quantitative radiomics features, derived from CT images of the whole-tumor ROIs on three-phrase, were extracted and selected by using ICCs, mRMR and LASSO methods. mRMR refers to Maximum Relevance and Minimum Redundancy, which is used to select the optimal features that are most relevant to the classification task but least redundant to each other. mRMR is an algorithm based on mutual information, similar to the Maximum Dependency algorithm. However, unlike Maximum Dependency algorithm, which is not applicable in the case of large number of features, mRMR is especially suitable for high-dimensional data space (25). After the redundant and irrelevant features were removed by mRMR, LASSO regression model was used to prevent overfitting of the selected radiomics features. The main advantage of LASSO method is that it does not compress the variable with larger parameter estimation, while the variable with smaller parameter estimation is compressed exactly to zero. The complexity of the model is controlled through a series of parameters, so as to avoid overfitting. Moreover, the parameter estimation of LASSO analysis has continuity, which is suitable for the model selection of high-dimensional data (26).

Finally, 14 features from three phases were identified as the most predictive subset of feature to construct the radiomics model, including one original first-order feature and eight wavelet-transformed features and five LoG-transformed features. Among the final selected features, the higher-order features filtered by wavelet and LoG filters were obviously superior to the original first-order and textural features. Chen et al. (16) investigated the role of CT-based radiomics to differentiate pelvic rhabdomyosarcoma from yolk sac tumors in children. Among the 10 features selected in their radiomics model based on each phrase, most of the selected features were wavelet-transformed features. Wavelet and LoG are both higher-order statistical methods imposing filter grids on the images, and could possibly reflect more information about vascularity and spiculation of the lesion (27). The principle of wavelet is to put a matrix of linear or radial “waves” on images, while LoG is mostly used to extract features from areas with coarse textural pattern (27). Besides, we evaluated the performance of radiomics features from two scanners, and the results showed that the performance of two signatures is different. One reason for this difference may be that radiomics features are correlated with different scanners from different manufactures, and the other reason may be that the sample size of patients scanned on Lightspeed VCT (GE Healthcare) was relatively too small.

The results of our study showed that radiomics models based on NP, AP and VP images can predict MYCN amplification in pediatric abdominal NB, while the performance of different machine learning radiomics models varies. The AUC in the training group among the Logistic, SVM, Bayes and Random Forest was 0.940, 0.940, 0.780 and 0.927, respectively, and the corresponding AUC in the testing group was 0.909, 0.909, 0.729, 0.851, respectively. The Logistic and SVM models have the best predictive performance with the same value of AUC. According to Delong test, there was no significant difference among the Logistic, SVM and Random Forest model, but all better than the Bayes model. In previous studies, researchers mostly chose one classifier to build radiomics model, and there is no consensus on the best-performing classifier method. Deist et al. (28) compared the performance of different classifiers (decision tree, random forest, neural network, support vector machine, elastic net logistic regression, LogitBoost) in predicting radiotherapy outcomes. In their study, Random forest and elastic net logistic regression performed better than other classifiers. Machine learning classifiers can be used to identify the best combination of radiomics features, while different algorithms have different advantages and disadvantages (29). Therefore, we should choose the optimal machine learning method with overall maximal predictive performance according to the specific clinical application.

In addition, we constructed a nomogram combining clinical parameters, imaging features and radscore. The variables including Shimada classification, VMA, infiltrating across midline and calcification were selected to build the nomogram with the most predictive signatures of radiomics. In this study, UFH was found to show significant correlation with MYCN amplification. This finding supported the previous study which also found that MYCN-amplified NBs were mostly categorized as UFH group (18). Besides, we found that the majority of NBs infiltrating across midline were MYCN-amplified, which is consistent with the finding of Wu et al. (8), but calcification in NB was found to be related to MYCN amplification. The nomogram had a superior predictive performance than using the clinical model alone, accompanied with an improved AUC value from 0.770 to 0.946 in the training group and 0.917 to 0.977 in the testing group. Besides, we found that the radiomics features used to construct the radiomics models were mostly derived from the NP and VP, so we developed Logistic radiomics model based on single VP. Then, we further evaluated and compared the predictive performance of the nomogram, Logistic radiomics VP model and Logistic radiomics three-phrase model. Although compared with radiomics model, nomogram did not significantly improve the prediction of MYCN amplification, they were both better than clinical model and radiomics model based on single venous phase, which demonstrated that the radiomics features are useful for predicting MYCN amplification and radiogenomics is expected to be involved in risk stratification in NB patients.

Despite our study showed that CT-based radiomics has the potential to predict MYCN amplification in pediatric abdominal NB, there were some limitations. First, this was a retrospective study, which may cause inherent selection bias, especially for those valuable absent clinical indicators that could potentially improve the performance of clinics-radiomics nomogram. Second, we only enrolled 172 patients in the present study because MYCN status has begun to be detected in recent years in our hospital. Previous literatures have shown that MYCN amplification usually occurs in about 20% of neuroblastoma. As a tertiary referral medical center, we have accumulated a certain number of MYCN-amplified cases over the past several years, and the inclusion of more cases will take some time in the future. Third, the CT scans of enrolled patients were performed on two scanners from different manufacturers in the study, from which the derived features have a certain influence on the predictive performance of radiomics models. Fourth, we only choose four common machine learning methods to build radiomics models, and the performance of other classifiers still needs to be evaluated.

## Conclusions

In conclusion, the CT-based radiomics signature is able to predict MYCN amplification of pediatric abdominal NB with high accuracy based on SVM, Logistic and Random Forest classifiers, while Bayes classifier yields lower predictive performance. Thus, one of these three machine learning methods should be the first consideration for researchers to construct predictive models for MYCN amplification of abdominal NB. When combined with clinical and radiographic qualitative features, the clinics-radiomics nomogram can improve the performance of predicting MYCN amplification. With the development of tumor molecular stratification, radiogenomics is expected to provide a promising method to characterize and predict molecular biomarkers noninvasively.

## Data Availability Statement

The original contributions presented in the study are included in the article/**Supplementary Material**. Further inquiries can be directed to the corresponding author.

## Ethics Statement

The studies involving human participants were reviewed and approved by The Ethics Committee of the Children’s Hospital Affiliated with Chongqing Medical University. Written informed consent from the participants’ legal guardian/next of kin was not required to participate in this study in accordance with the national legislation and the institutional requirements.

## Author Contributions

XC, HW, and LH contributed to conception and design of the study. KH, HD, LZ, TZ, and WY organized the database. HL performed the statistical analysis. HW wrote the first draft of the manuscript. XC and LH reviewed the manuscript. All authors contributed to manuscript revision, read, and approved the submitted version.

## Funding

This study was funded by Project Supported by Scientific and Technological Research Program of Chongqing Municipal Education Commission (Grant No.KJQN202000440); Basic Research and Frontier Exploration Project (Yuzhong District, Chongqing, China) (Grant No.20200155); and Science and Health Joint Medical Research Project (Science and Technology Commission and Health Bureau, Chongqing, China) (Grant No.2020FYYX128).

## Conflict of Interest

HL is an employee of GE Healthcare.

The remaining authors declare that the research was conducted in the absence of any commercial or financial relationships that could be construed as a potential conflict of interest.

## References

[1] Van Arendonk KJ Chung DH. Neuroblastoma: Tumor Biology and Its Implications for Staging and Treatment. Children (Basel) (2019) 6(1):12. 10.3390/children6010012 PMC6352222 30658459

[2] Cohn SL Pearson AD London WB Monclair T Ambros PF Brodeur GM. The International Neuroblastoma Risk Group (INRG) Classification System: An INRG Task Force Report. J Clin Oncol (2009) 27(2):289–97. 10.1200/JCO.2008.16.6785 PMC2650388 19047291

[3] Orr KE McHugh K. The New International Neuroblastoma Response Criteria. Pediatr Radiol (2019) 49(11):1433–40. 10.1007/s00247-019-04397-2 31620844

[4] Maris JM Hogarty MD Bagatell R Cohn SL. Neuroblastoma. Lancet (2007) 369(9579):2106–20. 10.1016/S0140-6736(07)60983-0 17586306

[5] Sokol E Desai AV. The Evolution of Risk Classification for Neuroblastoma. Children (Basel) (2019) 6(2):27. 10.3390/children6020027 PMC6406722 30754710

[6] Morgenstern DA Bagatell R Cohn SL Hogarty MD Maris JM Moreno L. The Challenge of Defining “Ultra-High-Risk” Neuroblastoma. Pediatr Blood Cancer (2019) 66(4):e27556. 10.1002/pbc.27556 30479064

[7] Yanishevski D McCarville MB Doubrovin M Spiegl HR Zhao X Lu Z. Impact of MYCN Status on Response of High-Risk Neuroblastoma to Neoadjuvant Chemotherapy. J Pediatr Surg (2020) 55(1):130–4. 10.1016/j.jpedsurg.2019.09.067 31685267

[8] Wu H Wu C Zheng H Wang L Guan W Duan S. Radiogenomics of Neuroblastoma in Pediatric Patients: CT-based Radiomics Signature in Predicting MYCN Amplification. Eur Radiol (2020) 31(5):3080–9. 10.1007/s00330-020-07246-1 33118047

[9] Lasocki A Rosenthal MA Roberts-Thomson SJ Neal A Drummond KJ. Neuro-Oncology and Radiogenomics: Time to Integrate? AJNR Am J Neuroradiol (2020) 41(11):1982–8. 10.3174/ajnr.A6769 PMC7658841 32912874

[10] Kuo MD Yamamoto S. Next Generation Radiologic-Pathologic Correlation in Oncology: Rad-Path 2.0. AJR Am J Roentgenol (2011) 197(4):990–7. 10.2214/AJR.11.7163 21940590

[11] Yan J Liu L Wang W Zhao Y Li KK Li K. Radiomic Features From Multi-Parameter MRI Combined With Clinical Parameters Predict Molecular Subgroups in Patients With Medulloblastoma. Front Oncol (2020) 10:558162. 10.3389/fonc.2020.558162 33117690 PMC7566191

[12] Iv M Zhou M Shpanskaya K Perreault S Wang Z Tranvinh E. MR Imaging-Based Radiomic Signatures of Distinct Molecular Subgroups of Medulloblastoma. AJNR Am J Neuroradiol (2019) 40(1):154–61. 10.3174/ajnr.A5899 PMC6330121 30523141

[13] Brisse HJ Blanc T Schleiermacher G Mosseri V Philippe-Chomette P Janoueix-Lerosey I. Radiogenomics of Neuroblastomas: Relationships Between Imaging Phenotypes, Tumor Genomic Profile and Survival. PloS One (2017) 12(9):e0185190. 10.1371/journal.pone.0185190 28945781 PMC5612658

[14] Vo KT Matthay KK Neuhaus J London WB Hero B Ambros PF. Clinical, Biologic, and Prognostic Differences on the Basis of Primary Tumor Site in Neuroblastoma: A Report From the International Neuroblastoma Risk Group Project. J Clin Oncol (2014) 32(28):3169–76. 10.1200/JCO.2014.56.1621 PMC4171360 25154816

[15] Meeus E Zarinabad N Manias K Novak J Rose H Dehghani H. Diffusion-Weighted MRI and Intravoxel Incoherent Motion Model for Diagnosis of Pediatric Solid Abdominal Tumors. J Magn Reson Imaging (2018) 47(6):1475–86. 10.1002/jmri.25901 PMC6001424 29159937

[16] Chen X Huang Y He L Zhang T Zhang L Ding H. Ct-Based Radiomics to Differentiate Pelvic Rhabdomyosarcoma From Yolk Sac Tumors in Children. Front Oncol (2020) 10:584272. 10.3389/fonc.2020.584272 33330062 PMC7732637

[17] Liu Y Zhang Y Cheng R Liu S Qu F Yin X. Radiomics Analysis of Apparent Diffusion Coefficient in Cervical Cancer: A Preliminary Study on Histological Grade Evaluation. J Magn Reson Imaging (2019) 49(1):280–90. 10.1002/jmri.26192 29761595

[18] Altungoz O Aygun N Tumer S Ozer E Olgun N Sakizli M. Correlation of Modified Shimada Classification With MYCN and 1p36 Status Detected by Fluorescence in Situ Hybridization in Neuroblastoma. Cancer Genet Cytogenet (2007) 172(2):113–9. 10.1016/j.cancergencyto.2006.10.005 17213019

[19] Pinto NR Applebaum MA Volchenboum SL Matthay KK London WB Ambros PF. Advances in Risk Classification and Treatment Strategies for Neuroblastoma. J Clin Oncol (2015) 33(27):3008–17. 10.1200/JCO.2014.59.4648 PMC4567703 26304901

[20] Iwatate Y Hoshino I Yokota H Ishige F Itami M Mori Y. Radiogenomics for Predicting p53 Status, PD-L1 Expression, and Prognosis With Machine Learning in Pancreatic Cancer. Br J Cancer (2020) 123(8):1253–61. 10.1038/s41416-020-0997-1 PMC7555500 32690867

[21] Khan S Naim S Bilwani R Salem A Gorlin D Muhammad A. Radiogenomics and Its Role in Lymphoma. Curr Hematol Malig Rep (2020) 15(3):211–24. 10.1007/s11899-020-00577-2 32430588

[22] Zhou M Leung A Echegaray S Gentles A Shrager JB Jensen KC. Non-Small Cell Lung Cancer Radiogenomics Map Identifies Relationships Between Molecular and Imaging Phenotypes With Prognostic Implications. Radiology (2018) 286(1):307–15. 10.1148/radiol.2017161845 PMC5749594 28727543

[23] Alessandrino F Shinagare AB Bosse D Choueiri TK Krajewski KM. Radiogenomics in Renal Cell Carcinoma. Abdom Radiol (NY) (2019) 44(6):1990–8. 10.1007/s00261-018-1624- 29713740

[24] Banerjee S Wang D Kim H Sirlin C Chan M Korn R. A Computed Tomography Radiogenomic Biomarker Predicts Microvascular Invasion and Clinical Outcomes in Hepatocellular Carcinoma. Hepatology (2015) 62(3):792–800. 10.1002/hep.27877 25930992 PMC4654334

[25] Peng H Long F Ding C. Feature Selection Based on Mutual Information: Criteria of Max-Dependency, Max-Relevance, and Min-Redundancy. IEEE Trans Pattern Anal Mach Intell (2005) 27(8):1226–38. 10.1109/TPAMI.2005.159 16119262

[26] Tibshirani RJ. Regression Shrinkage and Selection Via the Lasso. J ROY STA B (1996) 58(1):267–88. 10.1111/j.2517-6161.1996.tb02080.x

[27] Gillies R Kinahan P Hricak H. Radiomics: Images are More Than Pictures, They Are Data. Radiology (2016) 278(2):563–77. 10.1148/radiol.2015151169 PMC4734157 26579733

[28] Deist TM Dankers F Valdes G Wijsman R Hsu IC Oberije C. Machine Learning Algorithms for Outcome Prediction in (Chemo)Radiotherapy: An Empirical Comparison of Classifiers. Med Phys (2018) 45(7):3449–59. 10.1002/mp.12967 PMC6095141 29763967

[29] Erickson B Korfiatis P Akkus Z Kline T. Machine Learning for Medical Imaging. Radiographics (2017) 37(2):505–15. 10.1148/rg.2017160130 PMC5375621 28212054

