# Supplementary material for: CT-Based Radiomics Signature With Machine Learning Predicts MYCN Amplification in Pediatric Abdominal Neuroblastoma
Source: Front Oncol. 2021 May 24;11:687884. doi: 10.3389/fonc.2021.687884 (PMC8181422; doi:10.3389/fonc.2021.687884)
Supplement: Supplementary file 1 [file DataSheet_1.docx]

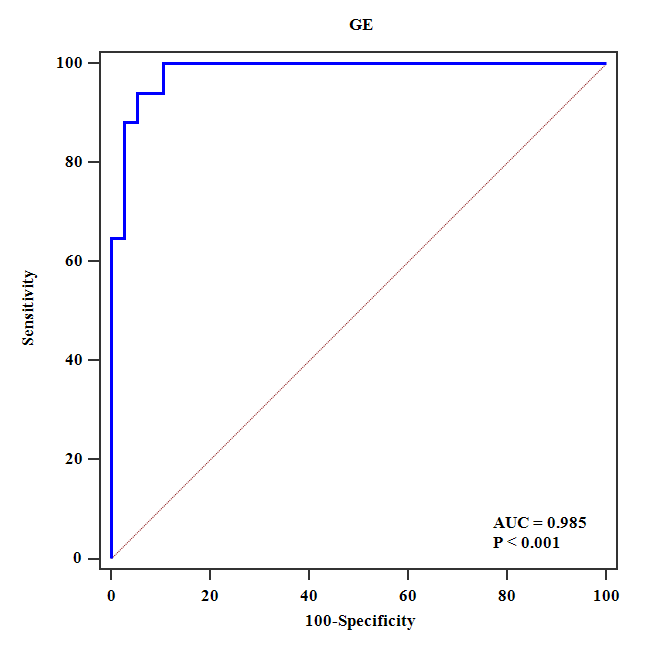

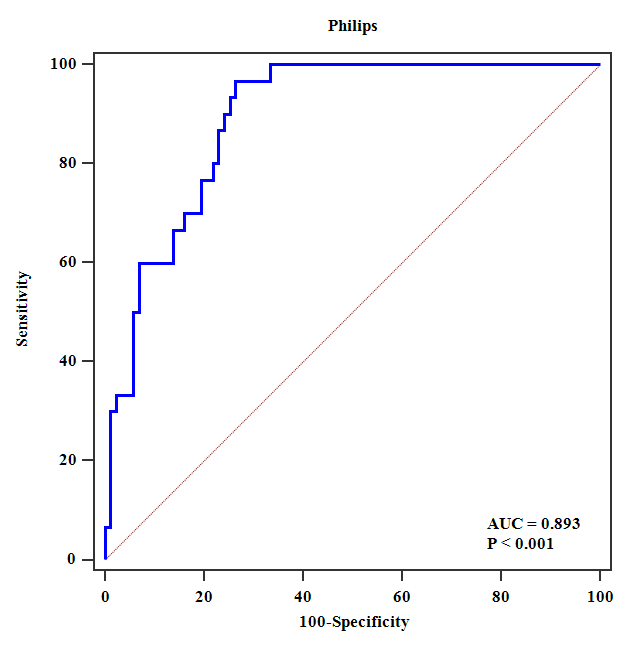


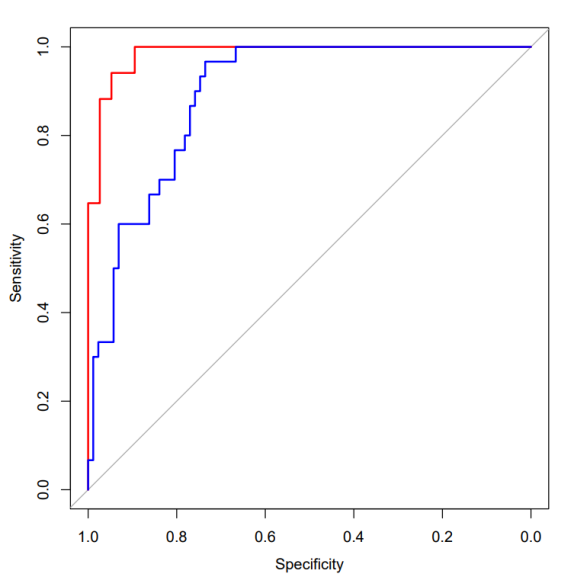


**Figure.1** The ROC curves of two radiomics features from two scanners (Lightspeed VCT (GE Healthcare, USA) and Brilliance ICT (Philips, Netherlands) ).

**Table 1** The comparison of radiomics features from two scanners.

|  | GE | Philips |
| --- | --- | --- |
| Area under the ROC curve (AUC) | 0.985 | 0.893 |
| 95% Confidence interval | 0.907-1.00 | 0.823-0.943 |
| Sensitivity | 1.00 | 0.9667 |
| Specificity | 0.8947 | 0.7356 |
| Delong test | 0.003748* | |

**Figure.2** The final formula of rad-score.
